# Structure and function of the nervous system in the stem of the siphonophore *Nanomia septata*: its role in swimming coordination

**DOI:** 10.1242/jeb.251974

**Published:** 2026-06-15

**Authors:** Tigran P. Norekian, Robert W. Meech

**Affiliations:** ^1^ Whitney Laboratory for Marine Biosciences, University of Florida, St Augustine, FL 32080, USA; ^2^ School of Physiology, Pharmacology and Neuroscience, University of Bristol, Bristol BS8 1TD, UK

**Keywords:** Hydrozoan colony, Nerve network, Swimming behaviour, Immunocytochemistry, Electrophysiology, Giant axon

## Abstract

The multiple swimming bells, or nectophores, of the colonial hydrozoan *Nanomia septata* are capable of coordinated avoidance swims in both forward and reverse directions. Individual nectophores also contribute to slower forms of swimming during foraging. Communication between a nectophore and the rest of the colony is at cone-shaped structures in the nectosome stem. The stem provides an attachment point for the nectophores and houses the simple nervous system responsible for their coordination. As revealed by immunocytochemistry, the nectosome stem has three main components: two giant axons, a distributed nerve network and a set of FMRFamide-immunoreactive nerve tracts. Whereas the nerve network is distributed throughout the stem, the nerve tracts link specific contralateral nectophores. Action potentials in the giant axons spread excitation rapidly along the stem, but their connection with individual nectophores is by way of the nerve network. Anatomical evidence suggests a location for the two pathways connecting the nerve network and the nectophore: one excites an epithelial impulse and leads to reverse swimming, and the other provides excitation for forward swimming by feeding into a ganglion-like cluster of nerve cells. The two-way exchange of neural information between the stem and the nectophore is by way of this terminal ganglion and a single nerve leading to a nerve ring at the nectophore margin. This work presents physiological evidence for mechanisms, such as facilitation and summation, operating within a multifunctional, bidirectional nerve network, responsible for coordinating epithelial and neural signals in an early-branching nervous system containing both condensed and distributed units.

## Teaser

**Summary:** A nervous system consisting of giant axons, diffuse nerve networks and FMRFamide-immunoreactive tracts coordinates multiple swimming bells to provide the *Nanomia septata* colony with well-organized foraging and escape behaviours.

## INTRODUCTION

**Fig. 1.**
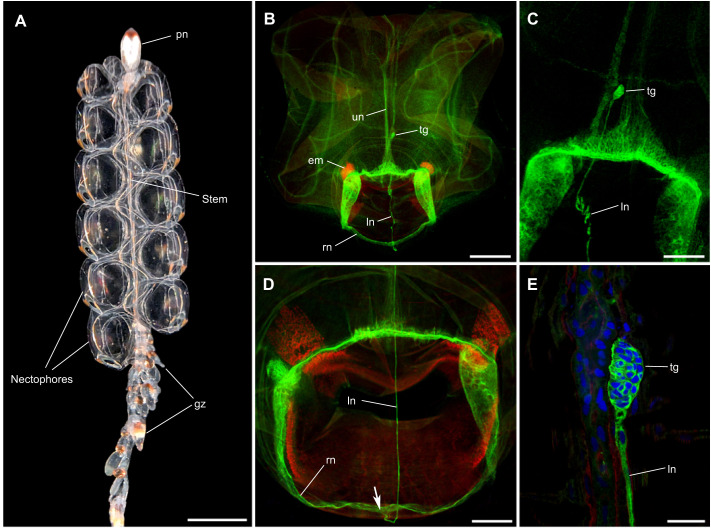
***Nanomia septata* and its nectophore structure.** (A) Living colony showing pneumatophore (pn), nectophores and gastrozooids (gz) attached to the central stem, visible through the transparent nectosome. (B) Entire isolated nectophore stained with anti-tubulin antibody (green) and phalloidin (red); both upper nerve (un) and lower nerve (ln) join the nerve ring (rn); endodermal muscles (em); the terminal ganglion (tg) is at the back of the nectophore where it contacts the stem. (C) The lower nerve ends in in the terminal ganglion. (D) Junction between the lower nerve and the nerve ring shown by an arrow; the circular and radial muscles of the velum are stained with phalloidin. (E) Terminal ganglion at high magnification; nuclei (blue) shown by DAPI labeling. Scale bars: (A) 5 mm; (B) 500 μm; (C,D) 200 μm; (E) 30 μm.

*Nanomia septata* is a colonial hydrozoan (Order: Siphonophorae) with many specialized zooids serving different organ-like functions, which can nevertheless act as an integrated swimming unit. At rest, the colony hangs vertically, supported by a gas-filled float (pneumatophore) at its anterior end. Below the pneumatophore are as many as 20 swimming bells, specialized zooids called nectophores, attached in columns on either side of an extended stem (Fig. 1A). New nectophores bud off from a growth zone behind the float (Totton, 1965; Siebert et al., 2015) and displace more mature bells further down the stem (Mackie, 1964). Together, the nectophores make up the nectosome. Attached to the stem below the nectosome is the siphosome with the colony's other specialized zooids. These include the hydroid-like gastrozooids, which trap prey using outspread tentacles, the male and female gonads, the defensive bracts, and the palpons specialized for digestion. The products of digestion are distributed by the stem (via the endodermal canal), which also houses the nervous system (Mackie, 1973; Mackie and Boag, 1963). This account explores the intriguing question of how the nervous system coordinates different colonial zooids to produce integrated swimming behaviours like those in unitary organisms.

The somewhat ungainly body form of *Nanomia* can perform surprisingly agile swimming manoeuvres. These consist of asynchronous swims by individual nectophores or blocks of nectophores, and synchronous swims by the entire nectosome (Mackie, 1964; Mackie et al., 1987). *Nanomia septata* spreads its tentacles during bouts of asynchronous swimming, whereas synchronous swimming is employed primarily for avoidance. Avoidance is of two kinds: a fast reverse swim after a stimulus in the float area, and a fast forward swim after a stimulus to the siphosome.

The ability to swim abruptly forwards or backwards is powered by contractions in a sheet of epithelial muscle (the myoepithelium) in the wall of each nectophore. When a nectophore bell contracts, seawater is forced outwards through an opening at the bell margin. Signals from the stem to the velum, a fringe of muscular tissue around the bell margin, configure it as a nozzle and regulate the size of its orifice (Mackie, 1964). The swim direction, backwards or forwards, depends on whether the nozzle points upwards or downwards. An upward configuration is associated with propagating signals in the ectoderm, which lead to the contraction of radial fibres on either side of the upper velum (Claus fibres; Claus, 1878; Mackie, 1964). The downward configuration during forward swimming is associated with neural signals in the lower nerve tract (Mackie, 1964) and the contraction of radial muscles in the lower velum (see ‘Role of radial muscles in determining the velum configuration’).

The giant axons and nerve networks in the stem of *Nanomia*, which contribute to the different forms of swimming, were originally revealed by electron and light microscopy (Mackie, 1973, 1978). The use of antisera against RFamide provided additional details about neural networks in the nectophores, the stem, and the muscular lamella that connects the nectophore with the stem (Grimmelikhuijzen et al., 1986). The structure of the nectophore nervous system was summarized by Norekian and Meech (2020) using an anti-tubulin antibody as a marker. It includes a nerve ring, a branching sensory upper nerve and a thin lower nerve, ending in the small terminal ganglion at the point of connection to the stem.

Here, we describe the structure of the stem nervous system using both tubulin and FMRFamide antibodies as markers, and highlight probable sites for chemical and electrical transmission, key pathways for forward and reverse swimming. Coordination of the nectosome requires cooperative interactions between individual nectophores communicating via their terminal ganglia and the stem nerve network. We monitored the exchange of information between nectophores and suggest roles for facilitation and summation.

## MATERIALS AND METHODS

### Animals

Adult specimens of *Nanomia septata* Mapstone, Mańko, Martell, Haddock & Hosia 2024 (originally *Nanomia bijuga* Delle Chiaje 1844) were collected from surface water at the dock of the Friday Harbor Laboratories, University of Washington, USA, and held in 3–4 litre containers at 10°C. As judged by their responsiveness to light and mechanical stimulation, animals survived in good condition for a week or more, but wherever possible, electrophysiology experiments were carried out on freshly collected specimens pinned to a Sylgard-coated Petri dish. Pins were placed near the float at the anterior of the animal and in the stem, just beyond the nectosome, at the posterior. The electrophysiology and kinematic analysis is based on 32 specimens collected in April–May 2025. Immunocytochemistry was carried out in the spring–summer seasons of 2022–2025.

### Immunocytochemistry

In this study, we used two markers to characterise the morphological structure of the nervous system in *N. septata*. Anti-tubulin immunoreactivity is a well-known and useful tool for identifying many of the neural elements in cnidarian nervous systems. Therefore, as a first marker, rat monoclonal anti-tubulin antibody was used (AbD Serotec, Bio-Rad, cat. no. MCA77G, RRID: AB_325003), which recognizes the alpha subunit of α-tubulin, specifically binding tyrosylated α-tubulin (Wehland and Willingham, 1983; Wehland et al., 1983). We have successfully used it to label the neural systems in *N. septata* nectophores (Norekian and Meech, 2020), the hydrozoan *Aglantha digitale* (Norekian and Moroz, 2020b) and several ctenophore species (Norekian and Moroz, 2016, 2019a,b, 2020a). In addition, FMRFamide (FMRFa)-like antigens have often provided valuable information about the nervous system (Grimmelikhuijzen and Spencer, 1984; Mackie et al., 1985; Satterlie, 2008, 2014; Satterlie and Eichinger, 2014), although those tested are in fact not specific for FMRFamide and instead appear to label the entire family of RFamide neuropeptides, distributed in diverse neural systems across the Metazoa (Grimmelikhuijzen and Spencer, 1984; Greenberg et al., 1988). Consequently, we used the anti-FMRFa antibody (rabbit polyclonal; Millipore, Sigma, cat. no. AB15348, RRID: AB_805291) not to locate FMRFamide specifically, but as an additional marker for neural elements. Anti-FMRFa antibodies and anti-tubulin antibodies therefore serve as complementary neuronal markers, and we used both to obtain a broad overview of the neural morphology of *N. septata*.

The muscles in the stem of *N. septata* contract strongly when placed in fixative, and so we relaxed the tissue by pre-incubation in high Mg^2+^ solution (1 part 0.3 mol l^−1^ MgCl_2_ and 2 parts filtered seawater) for 20 min prior to fixation. The nectosome was then fixed overnight in 4% paraformaldehyde in 0.1 mol l^−1^ phosphate-buffered saline (PBS). In this and in all subsequent incubations, the temperature was maintained at 4–5°C. Following incubation in the high Mg^2+^ solution, the nectosome maintained its natural form during fixation and most of the nectophores remained attached (nectophores are capable of autotomy unless treated with care). The preparation was subsequently washed 4–5 times in PBS over a period of several hours.

The fixed animals were dissected according to the task in hand. To investigate the stem, the stem was isolated by removing the nectophores. To investigate the connection between the nectophores and the stem, much of the nectophore bell was removed leaving only the area in direct contact with the stem. This improved the antibody penetration and gave better access to the contact area during the subsequent microscopy.

The dissected tissues were pre-incubated overnight in a blocking solution of 6% goat serum in PBS and then incubated for 48 h in the primary anti-tubulin antibody diluted with the 6% goat serum solution (final dilution ratio; ∼1:100). Following a series of PBS washes over a period of 8–12 h, the tissues were incubated for 24 h in secondary goat anti-rat IgG antibody, Alexa Fluor 488 conjugated (Molecular Probes, Invitrogen, cat. no. A11006, RRID: AB_141373), at a final dilution of ∼1:60. They were then washed for 12 h in PBS. Note that the concentration of antibodies used depended on the amount of tissue in the vial. A higher concentration gradient helped speed AB penetration, which was slow in stem whole mounts.

In immunocytochemical double-labelling experiments, we used both the anti-α-tubulin antibody and the antibody against FMRFa. After incubating in the blocking solution (6% goat serum in PBS), the tissues were placed for 48 h in the anti-FMRFa antibody diluted with 6% goat serum (final dilution ∼1:200). The anti-tubulin antibody was added to the same solution. After several PBS rinses over a period of 8–12 h, the tissues were placed for 24 h in secondary goat anti-rabbit IgG antibody, Alexa Fluor 568 (Thermo-Fisher, cat. no. A-11011, RRID: AB_143157), at a final dilution of ∼1:60. For the anti-tubulin antibody, we used goat anti-rat IgG antibody (Alexa Fluor 488 conjugated) in the same solution. After 12 h of washing, the tissue was mounted on glass microscope slides. As a control, we omitted either the anti-FMRFa antibody or the secondary antibody from the protocol; no labelling was detected in either case. Controls for the anti-tubulin antibody were as in Norekian and Meech (2020).

To label the muscle fibres, we used the well-known marker phalloidin (Alexa Fluor 488 phalloidin from Molecular Probes), which binds to F-actin (Wulf et al., 1979). After running the immunocytochemistry protocol, the tissues were incubated in phalloidin solution in PBS for 8 h at a final dilution of 1:80 and then washed in several PBS rinses for 12 h. The tissues were mounted on glass microscope slides in Vectashield mounting medium. The preparations were viewed and photographed using a Nikon research microscope Eclipse E800 with epifluorescence using standard TRITC and FITC filters, and a Nikon C1 Laser Scanning confocal microscope.

The fluorescent dye 4′,6-diamidino-2-phenylindole (DAPI) was used to label cell nuclei; DAPI binds to adenine-thymine (A-T) rich regions of double-stranded DNA, and is blue under UV light.

### Electrophysiology

Voltage steps from a bipolar electrode were used to elicit electrical signals in the nectophores and stem of *N. septata*. Voltage stimuli lasted 0.5–2.0 ms and were graded in amplitude. They may be seen as a ‘stimulus artifact’ at time 0 ms in some of the current records. Propagating signals, which had a threshold and an ‘all or nothing’ characteristic, were recorded using a fire-polished glass suction pipette. For stem recording, the tip of the pipette was introduced between neighbouring nectophores. Provided that the tip was advanced slowly, autotomy of nectophores was unusual. Once at the surface of the stem, gentle suction was applied to the pipette housing and a small area of stem wall was drawn onto the pipette tip. The tip diameter (0.32 mm OD; 0.22 mm ID) was selected to be wide enough for the recordings to provide a general overview of the electrical activity in the stem.

The electrical currents were amplified using a custom-made ‘loose patch’ clamp amplifier (Roberts and Almers, 1992). The current amplitude depended on the area of membrane drawn into the pipette and the proportion of current flowing to ground across the glass–stem interface. The voltage drop between the tip of the pipette and the input terminal of the amplifier depended on the current flowing across the stem surface and the resistance of the suction pipette.

### Analysis of externally recorded currents

The currents recorded at the stem surface were of two main types: a triphasic spike-like event and a more slowly developing ramp-like signal. Similar triphasic spikes have been recorded externally from other axons using a range of different techniques. Tasaki (1959) gives an example of the membrane current recorded from a short length of squid axon separated from the rest of the axon by two partitions; essentially the same arrangement as that employed here with the wall of the suction pipette providing the barriers at either side. The peak of the action potential, recorded at the same time with an intracellular micropipette, corresponded to the peak negative membrane current. Tasaki (1959) showed that, providing the axon had uniform properties, the membrane current was proportional to the second derivative of the propagating action potential. Experimental evidence to support this contention was supplied by Katz and Schmitt (1940), and Rushton (1937) provided a theoretical basis. Sub-threshold currents may be expected to spread from the stimulating to the recording site in a manner that depends on the space constant of the intervening conducting tissue. In a complex three-dimensional structure such as the stem, the spread of sub-threshold currents may be modelled using a delta function (Jack et al., 1975).

Contractions in the nectosome are accompanied by contractions in the longitudinal muscles of the stem ectoderm. These may be either fast, ‘twitch’ contractions, initiated by nerve impulses, or slow, postural contractions arising from propagating signals in the stem endoderm. The myoepithelium of the stem ectoderm is excitable but its electrical activity does not propagate independently. Instead, depolarizing currents can spread through ‘bridges’ of continuity across the mesogloea to the endoderm (Mackie, 1978). There they can summate, and when suprathreshold, indirectly excite the endodermal epithelium (Mackie, 1976), eliciting a slowly (0.4 m s^−1^) conducting impulse in the ‘S system’ (Mackie and Carré, 1983). Muscle spikes recorded externally from *Agalma*, another physonectid siphonophore, have durations of approximately 40 ms (Mackie, 1978) corresponding to those of intracellularly recorded fast events (Chain, 1979). These fast events appear on the rising phase of a slow depolarization lasting 1–2 s, similar to the SD wave recorded by Mackie (1978). Evidence for propagation within the endodermal epithelium is based on intracellular recordings from the stem of *Forskalia edwardsii* (Mackie, 1978). Endodermal impulses are evident in many of our records, frequently associated with ramp-like currents, but we have not examined their role in nectophore swimming in any detail.

### Kinematics

To aid us trace the origins of the different stem current signals we recorded the swimming responses of the *N. septata* colony to electrical stimulation. Images were collected at a high frame rate (240, 120 or 60 frames s^−1^) using an Apple iPhone mounted on a Wild microscope using a Tridaptor mount (MSM, Shenzhen, China). This allowed us to store the locations of the stimulating electrode and the recording pipette. The time of the stimulus was recorded by triggering a photodiode (located within the field of view) to flash at the same time. Occasionally, a flash covered two video frames; in this case, the frame with the brightest flash was taken to be the stimulus time. By matching the video with the electrical signal, we could distinguish between true current signals and movement artifacts. We could also match aspects of the behavioural response with the electrical signatures of the different stem components. Videos were converted into single frames using ‘Frame Grabber’ (Appcano LLC); we could find no evidence for ‘dropped frames’ in the data presented here. For presentation purposes, the contrast in selected frames was increased using Pixelmator (Apple Inc., Cupertino, CA, USA).

### Mg^2+^ anaesthesia

Seawater combined with isotonic MgCl_2_ (61.7 g l^−1^) is commonly used to reduce motor responses to external stimuli in invertebrate animals. In physiological studies on *Nanomia*, Mackie (1978) used an isotonic MgCl_2_:seawater solution in a ratio of 1:5, reporting that this concentration of MgCl_2_ did not affect impulse conduction in the stem but greatly reduced the amplitude of muscle twitch contractions. Even a 1:15 MgCl_2_:seawater solution ‘has a perceptible damping effect on twitch activity’ (Mackie, 1976). Previously, we have found that 10% isotonic MgCl_2_ in seawater was sufficient to suppress the contractions of nectophore striated muscle (Norekian and Meech, 2020) and we have used 1:10 isotonic MgCl_2_:seawater again here. Initially, the animals were highly sensitive to mechanical stimulation, giving coordinated avoidance responses, but after about an hour these coordinated responses became less evident. It is possible that this delayed effect may be because of diffusion barriers. Animals recovered their sensitivity after prolonged washing in normal seawater.

### Terminology used to specify the axes of the *N. septata* colony

**Fig. 2.**
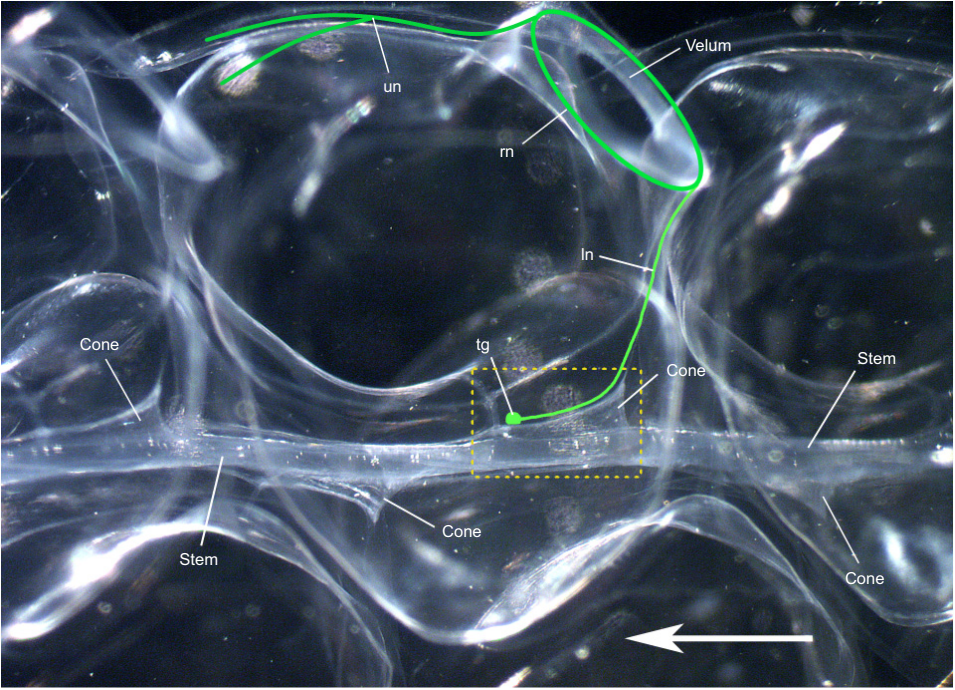
**The stem–nectophore attachment site.** The main elements of the nectophore nervous system shown schematically in green: rn, ring nerve; un, upper nerve; ln, lower nerve; tg, terminal ganglion. The area where signal exchange occurs between the nectophore and the stem is visible through the transparent tissue (see yellow dotted box). The arrow is aligned with the vertical axis of *N. septata* at rest and shows the direction of the pneumatophore; arrow length corresponds to 1.5 mm.

We use the term ‘anterior’ to describe the end of the colony with the pneumatophore. For isolated nectophores we use the terms ‘upper’ and ‘lower’; ‘dorsal’ and ‘ventral’ being retained as descriptors for the entire colony (see Haddock et al., 2005). Thus in Fig. 2 the nectophore nerves are identified as upper and lower.

## RESULTS

### Neuroanatomy

#### Overview of the nectophore nervous system

*Nanomia* nectophores are not only capable of synchronized contractions, driven by neural impulses in the stem, but also generate swims endogenously. Pacemaker neurons, located within an inner ring of nerves that encircle the bell margin (Fig. 1B; Norekian and Meech, 2020), are thought to be the source of this activity, as in other hydrozoans (Satterlie, 2014). On either side of the nerve ring are two column-shaped nerve plexuses and entering it at the top and bottom are two nerve tracts (Fig. 1B–D; Norekian and Meech, 2020). The extensively branching upper nerve appears to have a sensory role (Norekian and Meech, 2020), whereas the lower nerve (Fig. 1B,C) is unbranched until it arrives at, and merges with, the nerve ring (Fig. 1D). It contains a single giant axon (Mackie, 1973) and can be traced to the point of contact between the nectophore and the stem, where it terminates in a ganglion of 40–50 tightly packed neural cells (terminal ganglion; Fig. 1C,E; Norekian and Meech, 2020). Fig. 2 is an overall view of a mature nectophore and its connection to the stem; the positions of the main nerve elements are shown schematically.

#### The neural system in the stem revealed by tubulin immunoreactivity

**Fig. 3.**
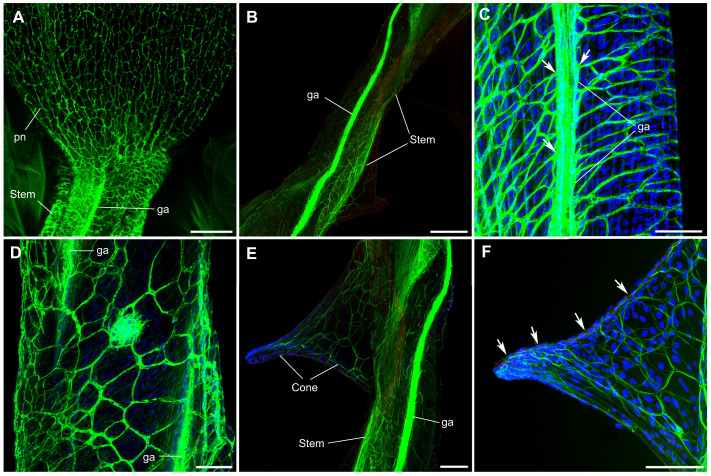
**Stem nervous system labelled with anti-tubulin antibody (green)**. (A) Junction between the stem and the pneumatophore (pn) showing origin of the giant axon (ga); the nerve network covering the pneumatophore surface merges with the stem nerve. (B) Giant axon running along the stem. (C,D) The nerve network covers the subepithelial layer in the stem and merges with giant axons at numerous points (arrows). (E) The cone-shaped structure, which serves as a nectophore docking site, contains an extension of the nerve network in the stem. (F) Higher magnification of the cone and its nerve network; arrows point to the nectophore attachment surface. Nuclei in C–F are stained blue with DAPI. See Fig. 2 for cone location within the nectosome. Scale bars: (A,B) 200 μm; (C–F) 100 μm.

**Fig. 4.**
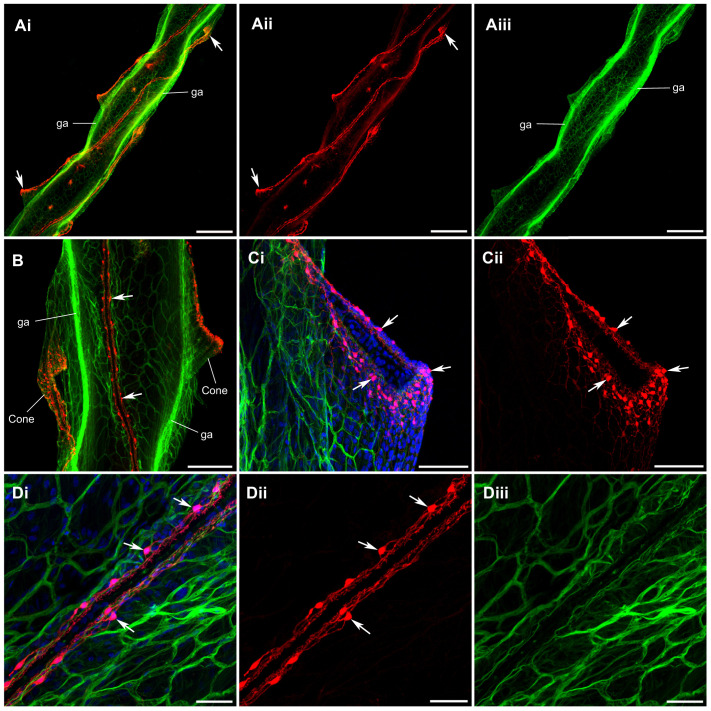
**Stem nervous system revealed by anti-tubulin antibody (green) and anti-FMRFa antibody (red)**. (Ai) Two lateral giant axons (ga) and their associated nerve network (green), together with FMRFa-ir neural tracts (red) connecting contralateral cones (arrows). Note that the connected cones are not immediate neighbours. (Aii) Red channel of Ai showing FMRFa IR only; (Aiii) green channel of Ai showing tubulin IR only. (B) Higher magnification of the stem showing giant axons, nerve network and neural tract (arrows), all running along the stem; note the FMRFa-ir neural loop at the tip of each cone. (Ci) Cone with its nerve network and FMRFa-ir neural loop at the cone tip. (Cii) Red channel of Ci showing FMRFa IR only; arrows show some of the numerous neural cell bodies. (Di) High magnification of the nerve network and the FMRFa-ir neural tract; arrows show some of the immunoreactive cell bodies. (Dii) Red channel of Di showing FMRFa IR only; arrows show immunoreactive cell bodies; (Diii) green channel of Di showing that the FMRFa-ir neural tract does not label with tubulin IR. See Fig. 2 for cone location within the nectosome. Cell nuclei in Ci,Di are stained blue with DAPI. Scale bars: (Ai–iii) 500 μm; (B) 200 μm; (Ci,ii) 100 μm; (Di–iii) 50 μm.

The tubulin immunoreactivity (IR) of the stem has two elements: a pair of giant axons and a subepithelial neural network connecting the axons with individual nectophores. The giant axons arise near the base of the pneumatophore (Fig. 3A) and run on either side of the stem (Figs 3A–E, 4A,B), so that each has a neighbouring column of nectophores. The axons are 40–50 μm in diameter (Fig. 3C) and in some areas their tubulin-immunoreactive (-ir) processes appear to split into separate fibres (Fig. 3C) as if each giant axon is derived from the fusion of many finer nerves. Electron microscopy (Mackie, 1984) shows that the axons have elongated nuclei distributed along their length, giving the impression of a syncytial system.

Merging with the giant axons at multiple places along the stem and located just under the outer epithelium (Fig. 3C) is a diffuse nerve network. Individual units vary in shape from triangular to octagonal and range in size from 10 to 100 μm across (Fig. 3D). The network covers the entire stem surface as well as extending into the pneumatophore (Fig. 3A). It also spreads into the apex of the cone-shaped structures shown in Fig. 3E,F. These cone-shaped protrusions serve as docking sites for individual nectophores and provide a platform for the neural connection between the central stem and the nectophore. Mackie (1964) described them as ‘a muscular pedicle through which passes an endodermal canal to the subumbrella’, while Grimmelikhuijzen et al. (1986) identified them as anchors or muscular lamellae.

#### Neural elements in the stem revealed by FMRFa immunoreactivity

In the stem of *N. septata*, FMRFa IR has revealed an additional element of the neural system, not seen with tubulin IR. As Fig. 4A shows, each cone-shaped nectophore attachment point is connected by a FMRFa-ir tract to another cone on the contralateral side – not its immediate contralateral neighbour, but the next one along. This double-stranded tract has an exceptionally strong FMRFa-ir signal and does not double-label with tubulin IR (Fig. 4B,D). It takes the form of a loop at the top of each cone, on the side contacting the nectophore (Fig. 4B,C). Both tract and loop consist of multiple thin processes and evenly distributed cell bodies (Fig. 3B–D).

**Fig. 5.**
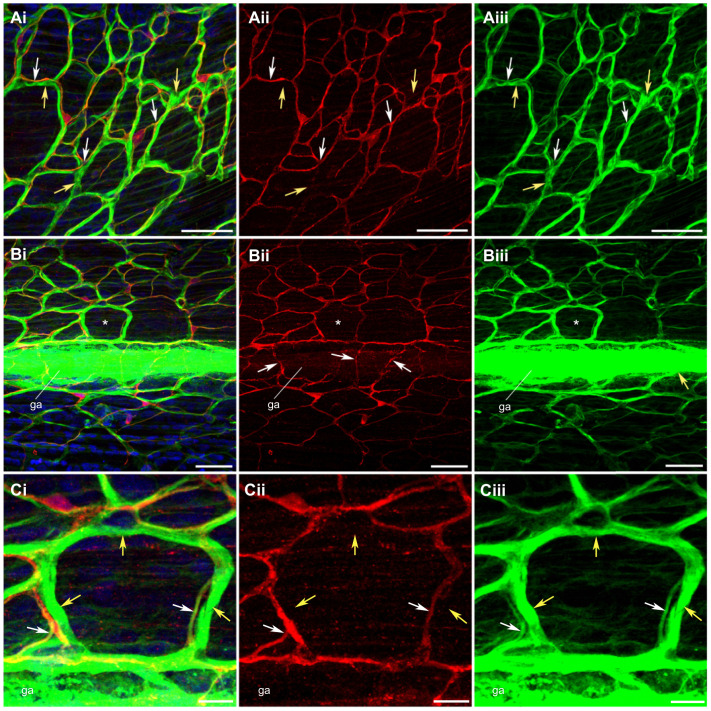
**Stem nerve network double-labelled with tubulin IR (green) and FMRFa IR (red).** (Ai) Double-labelled image. (Aii) Red channel with FMRFa IR only. (Aiii) Green channel with tubulin IR only; yellow arrows indicate thick processes with only tubulin IR; white arrows indicate processes with cross-reactivity for both tubulin IR and FMRFa IR. (Bi) Double-labelled image with giant axon (ga) and adjacent nerve network; asterisk shows pentagonal shaped neural unit. (Bii) Red channel, with FMRFa-ir processes (arrows) that cross the giant axon (unlabelled) rather than merging with it. (Biii) Green channel showing the giant axon labelled with tubulin IR; yellow arrow shows tubulin-ir threads merging with the giant axon. (Ci) Pentagonal shaped neural unit from Bi; (Cii) red channel with FMRFa IR only; (Ciii) green channel with tubulin IR only. Yellow arrows indicate thick neural threads with tubulin IR only; white arrows indicate processes with both tubulin IR and FMRFa IR. Scale bars: (A,B) 50 μm; (C) 12 μm.

A large part of the stem nerve network, including its thicker threads, has tubulin IR only and shows no FMRFa IR (Fig. 5A; yellow arrows). However, many thinner processes have both tubulin and FMRFa IR (Fig. 5A; white arrows). Thus, effectively, there are two nerve networks in the stem. They have matching polygonal structures and are located in the same focal plane (in optical sections ∼30 μm thick). However, although the thick fibres of the tubulin-ir network merge with the giant axons, the FMRFa-ir filaments can be seen crossing the giant axons (Fig. 5B) suggesting that they lie slightly below them. The situation in the cones is similar (Fig. S1) – part of the nerve network, including the thicker threads, has tubulin IR only, while part shows double-labelling. It is noticeable that the anti-FMRFa antibody stains the neural cell bodies particularly well, unlike the anti-tubulin antibody, which labels them rather poorly. Presumably, this reflects the distribution of the antigen molecules concerned.

#### The neural connection between nectophore and stem

**Fig. 6.**
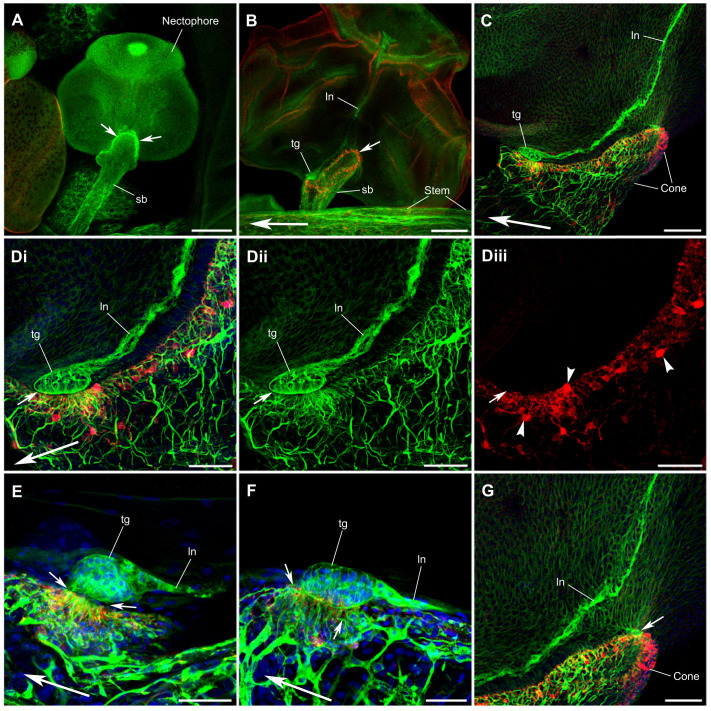
**Region of the stem–nectophore connection labelled with anti-tubulin IR (green) and anti-FMRFa IR (red).** (A) Early stage nectophore attached to the stem by an extended side-branch (sb). (B) Later stage nectophore showing lower nerve (ln) and terminal ganglion (tg) at the point of contact with the shortened stem branch (sb); arrow indicates the FMRFa-ir neural loop (red). (C) Mature nectophore connected to the stem via a short cone. (Di) Higher magnification of the terminal ganglion area from C; the terminal ganglion and cone are separated by a narrow cleft (arrow); many thin nerve fibres, both tubulin-ir and FMRFa-ir, come to the cone surface here. (Dii) Green channel of Di showing the nerve fibres opposite the nectophore terminal ganglion. (Diii) Red channel of Di showing FMRFa-ir cell bodies; labelling is absent from the terminal ganglion or lower nerve. (E) Side view of the contact between the terminal ganglion and the cone, showing thin nerve fibres at the cone surface, opposite the narrow cleft (arrows) that separates the cone from the terminal ganglion; nerve contains both tubulin-ir and FMRFa-ir elements. (F) View of the terminal ganglion, from the side and slightly above, showing that the nerve network gives rise to numerous fine processes (arrows) that project toward the terminal ganglion. (G) High magnification of C showing the area of contact between the nectophore epithelium and the cone surface (arrow); the outline of the epithelial cells is revealed by background tubulin-ir. D–F show different preparations. C–G are from the area shown by the yellow dashed box in Fig. 2. Cell nuclei in E,F are stained blue with DAPI. Scale bars: (A,B) 200 μm; (C) 100 μm; (Di–iii,G) 50 μm; (E) 40 μm; (F) 30 μm.

Early developing nectophores, arising near the pneumatophore, are connected to the stem via a thin extended branch (Fig. 6A). As new nectophores develop and increase in size, they move along the stem and the connecting branch shrinks. By the time the nectophore is fully mature, the connection has been reduced to a cone-shaped structure – the stem cone (Fig. 6B,C).

**Fig. 7.**
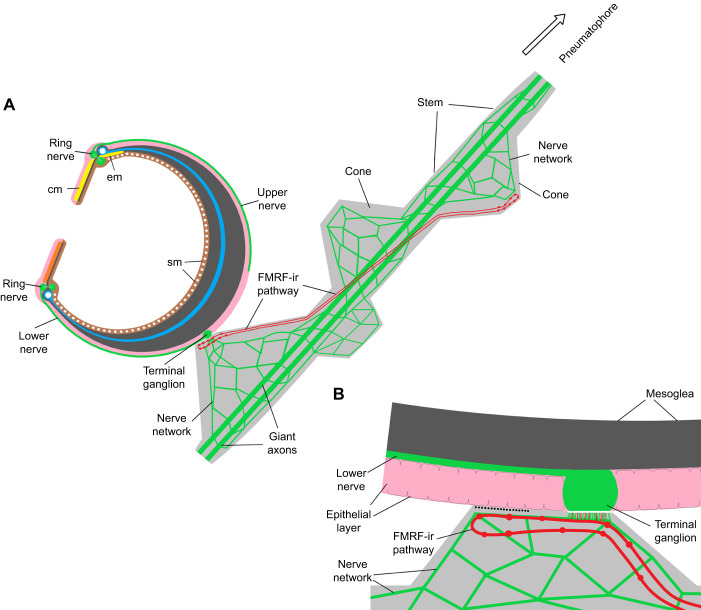
**Schematic representation of the neural contact between nectophore and stem.** (A) The stem neural system includes two giant axons, two tubulin-ir nerve networks and a system of FMRFa-ir, double-threaded, neural tracts between contralateral cones. The stem itself has two columns of cone-shaped protrusions that serve as docking stations for the nectophores. The terminal ganglion is at the attachment point; it is connected to the nerve ring, in the margin of the nectophore, by the lower nerve. Also shown are the Claus muscles of the velum (cm) and the muscles of the endoderm (em) that abut them (Norekian and Meech, 2020). (B) Details of the cone attachment area between nectophore and stem. The terminal ganglion of the nectophore is separated from the cone surface by a narrow cleft. Outside the terminal ganglion, the epithelial layer of the nectophore directly attaches to the cone. Here (black dotted line), electrical junctions may couple the nerve network of the cone with the epithelial conductance pathway in the nectophore.

Neural communication between a mature nectophore and the rest of the colony is via the lower nerve (Mackie, 1964, 1973) and its terminal ganglion (Fig. 6B,C). Cutting the lower nerve abolishes this communication. When nectophores are fixed with the stem in place, the lower nerve is seen to run parallel to the surface of the cone, bypassing almost the entire attachment area before ending in the terminal ganglion (Fig. 6D). The ectodermal epithelial layer that covers the nectophore's outer surface (Fig. 6D) is absent at the terminal ganglion itself, leaving a narrow cleft between the ganglion and cone surface (Fig. 6D,E). On the stem side of the cleft, many fine nerve fibres approach the cone surface (Fig. 6D–F). These thin fibres arise from thicker threads within the tubulin-ir nerve network and also from the FMRFa-ir double-threaded nerve tract. Outside the terminal ganglion, the epithelial layer of the nectophore is directly attached to the cone without an intervening cleft. Here, electrical junctions may couple the nerve network of the cone with the ectodermal epithelial conductance pathway in the nectophore. This epithelial pathway is instrumental in activating reverse swimming (Mackie, 1964). Fig. 7A is a schematic presentation of the data summarizing the structure of the stem–nectophore junction and highlighting (Fig. 7B) the proposed chemical and electrical synaptic connections.

### Physiological function and bell kinematics

#### Control of velum configuration by signals from the stem

Signals from within the *Nanomia* colony travel via the stem to individual nectophores and excite swimming (Mackie, 1964). Evidently, neural signals also travel in the opposite direction and spread excitation from a single nectophore through the entire nectosome. The nature of the transfer depends on the position of the nectophore in the nectosome (Mackie, 1964). A quantitative analysis on freely swimming *Nanomia* showed that electrical stimuli to anterior nectophores consistently elicited reverse swims, whereas stimuli to posterior nectophores elicited either forward swims or, more likely, a response restricted to the stimulated nectophore and its neighbours. Mid-way along the nectosome, in a ‘transitional’ region, stimulated nectophores could give either forward or reverse swims (Mackie, 1964).

**Fig. 8.**
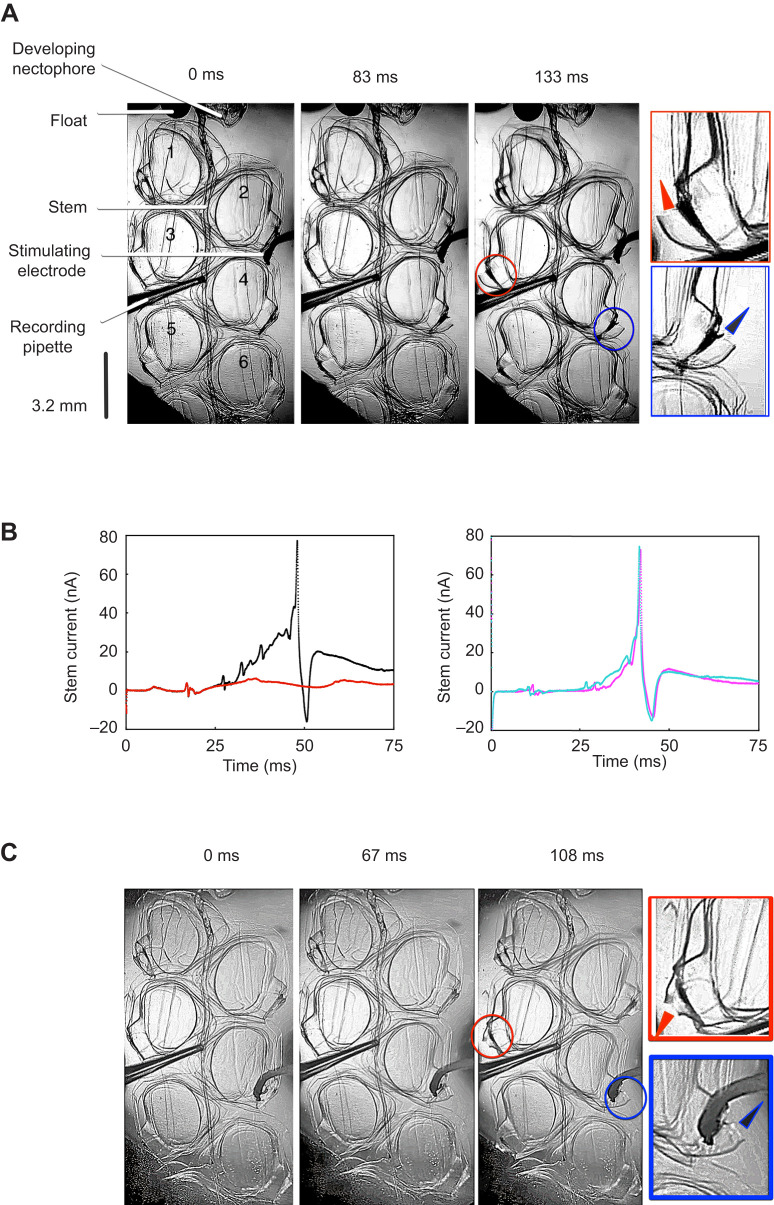
**Nectosome kinematics and electrical correlates.** (A) Nectosome with six nectophores (numbered); the stimulating electrode is on the upper surface of nectophore 4, and the recording pipette is attached to the stem between nectophores 3 and 4. Velum configuration at intervals after the stimulus (0, 83 and 133 ms); after 83 ms, the water leaving the bell of nectophore 4 had pushed the velum outwards, the unstimulated nectophores being unaffected, but by 133 ms, all nectophores were partly contracted with their vela deflected outwards. The nozzles in nectophores 3 and 4 are ringed in red and blue and enlargements are shown in boxes to the right; arrowheads indicate water flow. (B) Stem current after electrical stimulus to nectophore 4 at 0 ms. Left: superimposed current responses to successive stimuli; after the first stimulus (red trace), the stimulated nectophore contracted alone; after the second stimulus (black trace), all six nectophores contracted as in A. The black trace, visible once it diverges from the red trace, terminates with an ‘S’ potential. Right: superimposed current responses after stimulating electrode moved to near the nerve ring. Pink trace, record corresponding to the swim contraction in C. (C) Stimulating electrode on lower surface of nectophore 4, near its nerve ring. Velum configuration at different times after the stimulus (0, 67 and 108 ms); unstimulated nectophores have a forward swimming configuration. Enlargements of the vela of nectophores 3 and 4 are shown on the far right.

To follow the spread of excitation from individual nectophores to the rest of the colony, we video recorded changes in velum configuration. In most cases, the velum formed either an upward directed nozzle (for reverse swims) or a downward directed nozzle (for forward swims) as in Fig. 8A,C. However, we also saw instances where the nozzle configuration was symmetrical. In one preparation, electrical stimuli to an anterior nectophore gave generalized whole nectosome responses in five trials, and local responses in seven trials. During the generalized responses the nozzle was directed upward in the stimulated nectophore but downward in the rest of the nectosome. Similar mixed responses were obtained in four other preparations, although these preparations also responded with a uniformly upward configuration in some trials. In two preparations, trials were conducted on posterior nectophores; here, the most frequent generalized response was a downward configuration of the vela. These exploratory trials confirmed that the nectophore response depended on the position of the nectophore in the nectosome, but also raised questions about the effect of the location of the stimulating electrode and the influence of the rest-time duration between stimuli, questions that require further work.

Fig. 8A shows video frames of swims recorded after a stimulus to a transitional nectophore (4; numbering system shown in the left-hand frame). The stimulating electrode is seen to contact the nectophore upper surface. In that position, the velum exhibited a reverse swimming configuration in all six nectophores, in six out of 10 trials. In four trials, the configuration was also reverse, but excitation failed to spread beyond the stimulated nectophore. Overall, the stimulus response at a specific site was consistent in its configuration even if the spread was variable. Fig. 8C shows the response of nectophore 4 to a stimulus on its lower surface. In three trials, there was a reverse swim configuration in the stimulated nectophore, although the configuration was forward in the rest of the nectosome. Overall, suprathreshold stimuli to this transitional nectophore elicited generalized swim responses in 15 trials and localized responses in 41 trials. In 11 of the generalized responses, the vela had a reverse swim configuration as in Fig. 8A. In four trials, the velum in the stimulated nectophore had a reverse swim configuration but a forward swim configuration in the rest of the nectosome, as in Fig. 8C.

#### Role of radial muscles in determining the velum configuration

Mackie (1964) has described the role of the radially orientated Claus fibres in moulding the conformation of the upper velum during backwards swimming. Video recordings of the margin viewed head-on allowed us to determine the timing of the velar muscle contractions. Approximately 38 ms after the stimulus, there was a constriction of the velar orifice, probably caused by contraction of the circular muscles. At this point, water flow deflected the velum outwards, forming a symmetrical truncated cone (Gladfelter, 1972; Sutherland et al., 2019a). Approximately 12 ms later, contraction of the Claus fibres caused the water flow to be directed upwards by the velum's bulging lower rim (see enlarged side view in Fig. 8A, right).

The enlarged side view of the downwardly directed nozzle of nectophore 3 (Fig. 8C, right) highlights the role of the radial muscle fibres in the lower velum. During forward swimming, the Claus fibres retain their relaxed state; the lower rim of the velum actively contracts, the upper rim of the velum bulges outwards and water leaving the nectophore is deflected downwards.

#### Stem currents during local and propagated responses

The current elicited after stimulating nectophore 4 was recorded from the stem using a suction pipette (position shown in Fig. 8A). Fig. 8B (left) shows the superimposed current responses to two successive stimuli. After the first stimulus (red trace), the stimulated nectophore contracted alone, but after the second stimulus (black trace), all six nectophores contracted together. In the figure, the black trace is visible only after it begins a 30 nA ramp-like climb. The tri-phasic spike at the point of divergence is followed by three further tri-phasic events culminating in a large transient (∼100 nA peak to peak). Following Mackie (1964), we attribute this transient to currents associated with an action potential (‘S’ potential) in the epithelium lining the endoderm canal (see Materials and Methods). The progressive ramp-like current is attributed to the summation of events in the stem muscles (Mackie, 1964); the muscle epithelium is electrically coupled to the cells of the endodermal canal so that depolarizing events in the muscle summate and depolarize the endoderm to threshold. In Fig. 8B (right), the S potential had the same threshold as before, but the ripples and spikes on the rising phase are less distinct. The difference may reflect a small change in the position of the recording pipette.

#### Transfer of excitation from nectophore to stem: role of lower nerve and nerve network

**Fig. 9.**
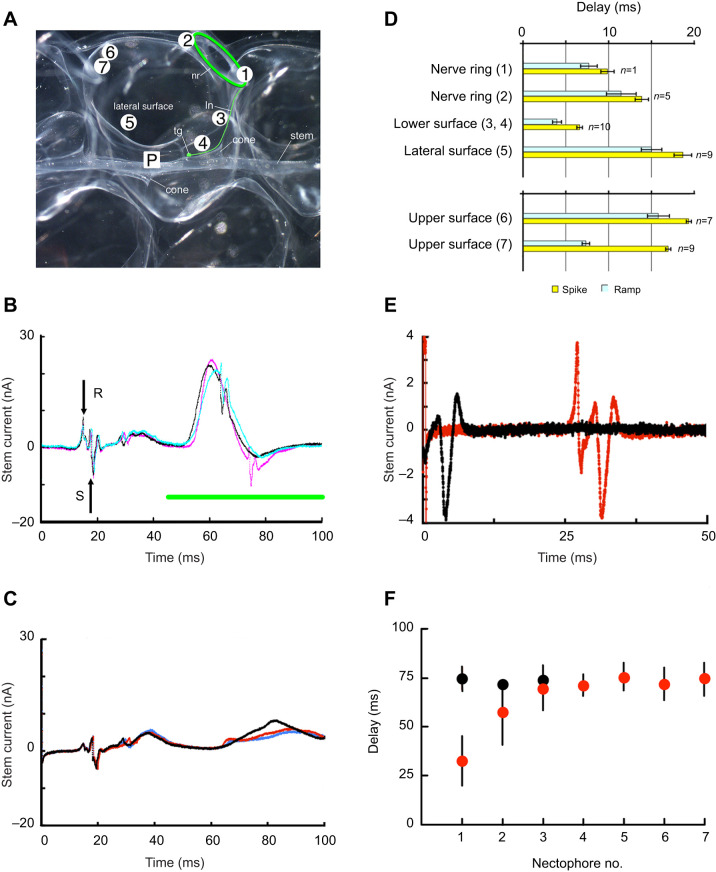
**Effect of stimulation site on stem currents.** (A) Nectophore and cone attachment area, showing the path (green) of the lower nerve (ln) from nerve ring (rn) to terminal ganglion (tg) and the different stimulation sites (1–7). P, pipette recording site. (B) Superimposed current records after three successive stimuli to site 6, at 0 ms, showing ‘ramp’ (R) and ‘spike’ (S) components. The green bar shows the timing of nectophore contractions. The records are associated with backward swim configuration of the stimulated nectophore. (C) Superimposed current records after three successive stimuli to nectophore site 5 at 0 ms. The records are associated with forward swim configuration of the stimulated nectophore. (D) Mean delay (±s.d.) from stimulus to ramp, or spike, at different stimulus locations (1–7). (E) Superimposed currents from another preparation after a stem stimulus (black) and a stimulus to a nearby nectophore (red); stem recording site is unchanged; deflection at 0 ms is the stimulus artefact. (F) Delay (±s.d.) to just discernible velum contraction in different nectophores following stimulation of a posterior nectophore (red) or the nearby stem (black).

The spread of excitation from the nectophore to the stem was examined by stimulating different sites on the nectophore surface, the position of the recording site on the stem remaining fixed. A suitable recording site, near the point of attachment of the nectophore to the stem, was obtained by gently pushing the pipette between the stimulated nectophore and its neighbour. In one trial, a nectophore was stimulated at three different sites, each equidistant (6.5 mm) from the recording pipette. The current response had a complex shape with a ramp-like early component (R; Fig. 9B). When the stimulating electrode was on the upper surface of the nectophore, the mean±s.d. time between the stimulus and the top of the ramp was 11.5±0.5 ms (*n*=3); stimulating the lateral surface produced a similar delay (11.8±0.1 ms, *n*=3), but when the stimulus was near the lower nerve (see Fig. 2), the delay was significantly less (4.5±0.7 ms, *n*=3). The combined values obtained from this and another preparation were 4.8±1.7 ms (*n*=24) for stimulations near the lower nerve and 10.7±1.4 ms (*n*=38) for stimulations at other sites.

Fig. 9A–D summarizes data from a third preparation in which stimuli were repeatedly applied to specific locations on the nectophore surface. The currents elicited were recorded at a fixed point (P) located on the stem near the nectophore attachment point. Fig. 9A shows the path of the lower nerve and the numbered positions of the stimulating electrode. Fig. 9B shows three superimposed current records obtained with the stimulating electrode touching the nectophore's upper surface (position 6). The early current transients, identified as a ramp (R) and a triphasic spike (S), were recorded before any visible contraction in either the velum or the nectophore (shown by the green bar). A lower amplitude triphasic event riding on a slower deflection was also recorded, as well as a large (20–25 nA amplitude) slow wave that peaked at 60 ms. A fast triphasic spike appeared on the falling phase of the slow wave. All three records were associated with a reverse swim configuration in the stimulated nectophore.

When the stimulating position was changed to one nearer the recording electrode on the nectophore's lateral surface (position 5), the early currents once again consisted of an initial ramp followed by a spike (Fig. 9C). The slower ramp with its low amplitude triphasic spike was also present; but the large slow wave was absent. Note that in position 5, all stimuli produced a forward swim configuration in the stimulated nectophore. For any given stimulation site, the early currents were consistent in shape, although responses from different sites had significant timing differences (Fig. 9D). As before, the delay from stimulus to ramp was shortest with the stimulating electrode near the lower nerve. The delay between the stimulus and the spike was also short in this position. The longest delays were when the stimulating electrode was furthest from the lower nerve, on the nectophore's upper or lateral surfaces.

The delay between the stimulus and the current response in the stem was reduced if the stem was stimulated directly. In Fig. 9E, the simple triphasic current evoked by a stimulus to the stem (black trace) is compared with the combined ramp and spike that followed a stimulus to the nectophore (red trace). The two stimulation sites were approximately equidistant (3.4 mm) from the recording site (similar position to that in Fig. 8). The calculated conduction velocity was 0.9 m s^−1^ (at 10°C).

The spread of excitation from a single stimulated nectophore to the rest of the nectosome was assessed from video records of coordinated swims. Fig. 9F shows the delay (mean±s.d.) between the stimulus and the earliest perceptible velar contraction, in each of the mature nectophores in the nectosome. The red data points show averages (*n*=7) in which the most posterior nectophore (1 in Fig. 9F) was stimulated; in each case, the velum had a forward swim configuration. In the stimulated nectophore, the earliest velar movement depended on the position of the stimulating electrode on the swimming bell (16–44 ms after the stimulus). The delay from stimulus to the earliest movement in the neighbouring velum (nectophore 2) was either <42 ms (*n*=3) or >66 ms (*n*=4). For nectophores 4–7, the mean delay was 73.4 ms (*n*=27; range 66.6–87.5 ms).

Fig. 9F also shows the delay to velum contraction when the stem was stimulated directly near nectophore 1 (black data points); in the case of nectophore 2, the mean±s.d. delay was 71.9±2.1 ms (*n*=4). When nectophore 1 was stimulated directly, the contraction delay in nectophore 2 (mean±s.d., 57.7±16.9 ms) was significantly less than that observed after the stem stimulus (*P*=0.036, *t*=−2.2).

#### Stem currents during stimulated and spontaneous swim contractions

**Fig. 10.**
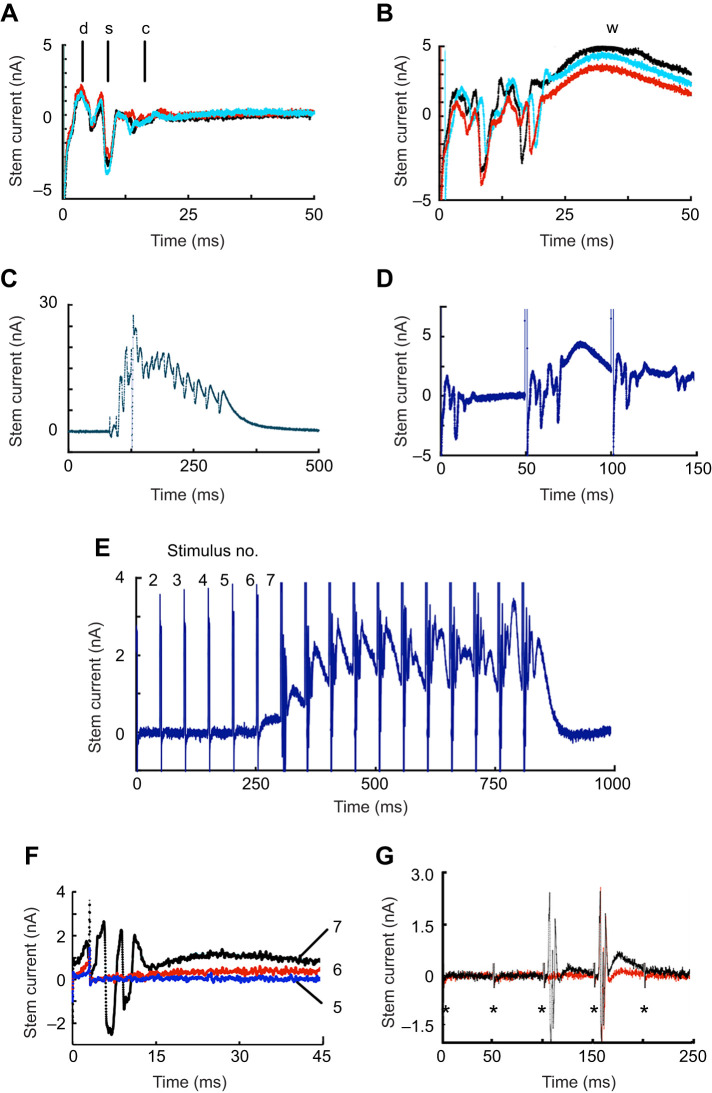
**Evoked electrical currents recorded in the stem.** (A) Stem currents following a stimulus to the stem (at 0 ms). The three superimposed records show a broad deflection (d), a single triphasic ‘spike’ (s) and a late complex (c); there were no swim contractions. (B) As A, but each stimulus elicited two triphasic spikes, a late slow wave (w) and a swim contraction in the nectophore nearest the stimulating electrode. (C) Stem currents showing spikes and slow waves during a spontaneous swim. (D) Stem currents following repeated stem stimulation (marked by stimulus artifacts, 50 ms apart); the second stimulus elicited two triphasic spikes and a late slow wave. There was a swim contraction in a nearby nectophore. (E) Repeated subthreshold stem stimulation (stimuli 50 ms apart) showing a change in excitability. (F) Currents associated with stimuli 2–4 in E were averaged and subtracted from the current responses to stimuli 5, 6 and 7. The resulting currents (labeled) are shown superimposed. (G) Potentiated slow wave following repeated stimuli (asterisks): during trial 1 (red), stimulus 4 elicits a single triphasic current followed by a low-amplitude slow wave; during superimposed trial 2 (black), stimulus 3 elicits a single triphasic current with a low-amplitude slow wave resembling that following stimulus 4 in trial 1; stimulus 4 again elicits a single triphasic current but the slow wave is markedly potentiated.

Although a suprathreshold electrical stimulus to the stem can elicit a rapidly conducting current spike, a coordinated swim movement does not necessarily accompany the spike. Fig. 10A,B shows currents recorded from the stem, in the nectosome's transitional zone; the recording site was 3 mm from the stimulating electrode, in the posterior direction. Following the stimulus artifact (at 0 ms), and a positive-going deflection, there was a triphasic ‘spike’ and a series of low amplitude waves (as in Fig. 9B,C). The conduction velocity of the spike, estimated from the time to its negative-going peak, was approximately 33 cm s^−1^ (at 10°C). Stimuli that generated a single spike (Fig. 10A) failed to elicit any nectophore contraction. However, a similar stimulus that generated a double spike (Fig. 10B) produced contraction of the nectophore closest to the stimulation site. The second spike was followed by a slow wave with a peak at approximately 30 ms.

During spontaneous nectophore contractions, flurries of spikes and slow waves were recorded from the stem (Fig. 10C), and the effect could be reproduced by repeated stimuli as shown in Fig. 10D. The stimuli were 50 ms apart and the second stimulus elicited a double spike and a contraction in the nearby nectophore. As in Fig. 10B, the double spike was followed by a slow wave.

#### The role of facilitation in the nectophore stem

Mackie (1964) found he could evoke asynchronous forward swimming by ‘prolonged gentle agitation of the siphosome’. It is possible that each stimulus leaves behind an after-effect, which facilitates the transmission of the next stimulus, a form of ‘facilitation’ (Pantin, 1935; Satterlie, 2002; Anctil, 2015). To examine this possibility, we recorded stem currents during repeated low-level stimulation. We found that the slow wave shown in Fig. 10B, which consistently followed pairs of triphasic spikes, could also be elicited by repeated subthreshold stimuli. In Fig. 10E there was little visible long-term change in the current baseline until after stimulus 6. For Fig. 10F, the currents associated with stimuli 2–4 were averaged and subtracted from the responses to later trials. After stimulus 6, there was a slowly developing positive deflection of the baseline; after stimulus 7, this deflection resembled a slow wave and was preceded by a triphasic spike. In another set of trials, sequential triphasic spike-evoking stimuli were found to elicit a potentiated slow wave (Fig. 10G).

## DISCUSSION

Neural, endodermal and muscle systems all contribute to the electrical currents recorded from the stem of *Nanomia* following brief voltage stimuli (Mackie, 1978). Here, we focused on the neural currents as a link between *Nanomia'*s neuroanatomy and its behaviour. For example, the way in which the colony generates coordinated forward or backward swimming has long been a puzzle. Pairs of action potentials in the giant axons give rise to reverse swims but a second, more slowly conducting, stem system may be involved in forward swimming (see Mackie, 1964, 1973). The presence of a double-stranded nerve network, an FMRFa-ir neural tract and a ganglionic gateway between the stem and the nectophore, may account for some of the complexity involved.

The association between the giant axons and the stem nerve network has been demonstrated by phase contrast and electron microscopy (Mackie, 1973, 1978) and by the spread of injected dye (Grimmelikhuijzen et al., 1986). This dye coupling may reflect electrical coupling, and if so, could explain how excitation can jump damaged sections of giant axon (Mackie, 1973). We found that the stem nerve network has two overlapping components: one, with tubulin IR only, has thick threads; the other, with thinner threads, double labels with both tubulin and FMRFa antibodies (Fig. 5). The thicker threads merge with the giant axons at sites along the whole length of the stem, whereas the thinner double-labelled elements seem to cross the giant axons without merging (Figs 3 and 5). The existence of two networks corresponds to the earlier observation, under phase contrast, that the stem nerve network seems double-stranded (Mackie, 1978). We find that it has two overlapping components: one, with tubulin IR only, has thick threads; the other, with thinner threads, double-labels with both tubulin and FMRFa antibodies (Fig. 5). The thicker threads merge with the giant axons at sites along the whole length of the stem, whereas the thinner double-labelled elements seem to cross the giant axons without merging (Figs 3 and 5).

### Coordinated forward and reverse swimming

During forward swimming, signals travel from the stem to the nectophore along the lower nerve tract (Mackie, 1964, 1973), presumably via the terminal ganglion, which is where the lower nerve originates (Norekian and Meech, 2020; Fig. 6D–F). Signals passing from the cone nerve network to the terminal ganglion must cross a narrow cleft that lies between the two. Nerve processes approach the terminal ganglion here but do not appear to cross into it, and so the transmission mechanism has yet to be determined. Once at the nectophore nerve ring, excitation spreads to the velum and the circular swimming muscles of the subumbrella. Contraction of the subumbrella generates thrust while the velar muscles mould the velum into a backward pointing nozzle (Fig. 8C; red box).

Reverse swims, seen most commonly when the float of *Nanomia* float contacts the air–water interface, depend on signals propagating within the exumbrellar ectoderm (Mackie, 1964). However, a nerve pathway must excite the nectophore ectoderm because the stem ectoderm is known to be inexcitable (Mackie and Carré, 1983). Presumably, this neural–epithelial transmission occurs in the cone area where the nectophore meets the stem, but away from the terminal ganglion. Here, there is a diffuse association between the nerve network and the nectophore's ectodermal epithelium, which might provide an electrical pathway between the two (Fig. 6C,G). Future work might search the area for gap junctions or test for dye coupling.

Ectodermal impulses travel to Claus fibres on the left and right of the upper velum, causing them to contract. Excitation of the Claus fibres is indirect and involves a neural intermediate located within the nerve ring (Norekian and Meech, 2020). Contracted Claus fibres hold the upper velum so that when seawater is forced from the bell, the lower velum bulges outwards, forming an upwardly pointing nozzle (Fig. 9A). This means that during a reverse swim, signalling in the lower nerve must be absent, perhaps inhibited in some way. The absence of inhibition may generate a symmetrical nozzle configuration in some instances.

### Asynchronous swimming and the spread of excitation between nectophores

Although gentle, mechanical stimulation of the siphosome can evoke asynchronous forward swimming (Mackie, 1964), it more generally arises spontaneously, probably from pacemaker activity in the nectophore nerve ring as with other hydrozoans (Satterlie, 2002). We suggest that the spread of excitation from one nectophore to its neighbours is by way of the terminal ganglion because stimulating the nectophore at sites near the lower nerve tract excites signals in the stem with the least delay.

To examine the further spread of excitation, we measured the delay to just detectable velum movement for each nectophore in the nectosome. In directly stimulated nectophores, the delay was 16 to 44 ms (Fig. 9F) compared with delays of either <42 ms (*n*=3) or >66 ms (*n*=4) in the nearest neighbour, a spread of values that deserves further examination. In more distant nectophores, the delay was more consistent (mean 73.4 ms; *n*=27; range 66.6 to 87.5 ms). We conclude that communication between the terminal ganglion and the stem nerve network involves a significant delay, but excitation then spreads rapidly through the remaining nectosome.

### The facilitation barrier

Mackie (1978) could induce swimming by stimulating the stem twice within a 20 ms interval, and suggested that there was a ‘facilitation barrier’ between the stem and the motor neurons in the nectophore. We explored the characteristics of the stem response to repetitive stimulation using different stimulus frequencies. In Fig. 10E, the axon reached its action potential threshold after seven stimuli. However, the earlier subthreshold stimuli elicited local responses that may contribute to action potential initiation. After stimulus 6, there was a slowly rising positive current that reached a broad peak at approximately 45 ms; after stimulus 7, the slowly rising current had an increased amplitude and a shorter time to peak.

Repetitive spiking recorded from the stem had a frequency of approximately 6 impulses per second (Mackie, 1976). Similar low-frequency impulses in crustacean axons have been modelled using modified Hodgkin/Huxley equations with the addition of a transient potassium current (called *I*_A_; Connor et al., 1977). To provide a satisfactory model, *I*_A_ must be susceptible to inactivation by a maintained low-level depolarization. A transient potassium current of this kind in *N. septata* axons would be inactivated by a local response like that shown in Fig. 10F, which would clear the way for a supra-threshold response. Such currents are present in another hydrozoan axon (Meech and Mackie, 1993).

### Role of summation

Normally, stimulation of an anterior nectophore produces a reverse swim configuration throughout the nectosome. However, we did observe a change in response following autotomy of one of the nectophores. Autotomy did not affect the transmission of excitation down the stem, but it did interrupt the signal for reverse swimming. This led us to suppose that excitation of the exumbrellar ectoderm (necessary for reverse swimming) is a cooperative process with contributions from each nectophore in turn. With high-speed propagation along the stem, much of the nerve network would be excited together and be able to exert a cooperative influence. A reduction in total current resulting from loss of a nectophore by autotomy would cause a rapid spatial decay of current, leading the ectoderm to fail to reach threshold (see Tomita, 1967; Jack et al., 1975).

This kind of explanation might account for the finding of Sutherland et al. (2019b), who observed that in freely swimming *Nanomia bijuga*, the velum can, on occasion, switch from a forward to a reverse configuration, midway through a swim. We have confirmed this observation in an electrically stimulated transitional nectophore: a forward swim in its contralateral neighbour became converted to a reverse configuration partway through its contraction. If cooperative activity in the nerve network is necessary for the ectodermal epithelium to reach its firing threshold, late contributions from distant neighbours might induce a delayed ectodermal excitation in the contralateral one.

It is possible that during forward swimming, excitation travels along the stem at a speed that fails to generate a cooperative response sufficient to activate the ectodermal system. However, we have observed that a stimulus to a posterior nectophore that produces forward swims in the rest of the nectosome may be followed some 600 to 1200 ms later by a backward swim. It is as if the first impulse can provide a primer for the second, enabling it to activate the ectodermal system. Fig. S3 provides a hypothetical basis for the exploration of possible cooperative effects.

### FMRFa-ir-labelled network and neural tract

The functional significance of the FMRFa-ir-labelled network in the stem of *N. septata* is unclear. FMRFa-ir is widespread in cnidarian neuronal structures, including the sensory epithelium and ocellar nerve cells in the hydrozoan *Polyorchis* (Grimmelikhuijzen and Spencer, 1984) and sensory cells in cubomedusae and scyphomedusae (Eichinger and Satterlie, 2014; Satterlie and Eichinger, 2014). In *N. septata*, there is an extensive network of FMRFa-ir cells around the nectophore nerve ring, some of which may be sensory (Fig. S2; Grimmelikhuijzen et al., 1986).

The FMRFa-ir neural tract in the stem connects each nectophore cone with the cone of its contralateral neighbour but one. The precise nature of this organization, which specifically avoids a direct connection between the cones of nearest neighbours, is highly intriguing. It seems likely that its activity influences the pattern of swimming in some way, but whether it promotes or inhibits excitation remains uncertain. We recorded no instance of an electrical stimulus to one nectophore spreading excitation to distant contralateral neighbours without exciting intervening ones, and propose that the FMRFa-ir neural tract prevents a spontaneous asynchronous swim during feeding becoming a synchronous escape swim. There is a precedent for this: swimming in many hydrozoans is inhibited during feeding, and in *Aglantha digitale* the nerve circuits involved are FMRFa-ir labelled (Mackie et al., 2003, 2012; Mackie and Meech, 2008). Thus, the FMRFa-ir neural tract may be part of ‘the blocking or filtering mechanism’ which regulates the traffic between individual zooids and the rest of the colony (Mackie, 1978).

## Supplementary Material

10.1242/jexbio.251974_sup1 Supplementary information
